# Acute Toxicity Testing of the Tire Rubber–Derived Chemical 6PPD‐quinone on Atlantic Salmon (*Salmo salar*) and Brown Trout (*Salmo trutta*)

**DOI:** 10.1002/etc.5487

**Published:** 2022-11-07

**Authors:** Anders Foldvik, Fedor Kryuchkov, Roar Sandodden, Silvio Uhlig

**Affiliations:** ^1^ Department of Salmonid Fishes Norwegian Institute for Nature Research Trondheim Norway; ^2^ Toxinology Research Group, Norwegian Veterinary Institute Ås Norway; ^3^ Section for Environmental and Biosecurity Measures, Norwegian Veterinary Institute Trondheim Norway; ^4^ Nordic Institute of Dental Materials Oslo Norway

**Keywords:** 6PPDQ, 6PPD‐quinone, aquatic toxicology, contaminants of emerging concern, ecological risk assessment, toxic effects, runoff, *Salmo*

## Abstract

Recent identification of 6PPD‐quinone as the chemical causing acute toxicity in coho salmon has led to substantial concern regarding the toxicity of this contaminant for other aquatic species. Environmental occurrence of 6PPD‐quinone is probably high, because it is an oxidation product of a common tire rubber additive. Research on 6PPD‐quinone toxicity in fish has revealed a rather unusual pattern, with closely related species exhibiting responses ranging from extreme sensitivity to no effect. Of 11 previously studied fish species, 6PPD‐quinone was toxic to four. The species‐specific toxicity of 6PPD‐quinone complicates urgently needed environmental risk assessment. We investigated the acute toxicity of 6PPD‐quinone in Atlantic salmon and brown trout alevins (sac fry). These species have previously not been tested for sensitivity to 6PPD‐quinone. The fish were exposed in static conditions in eight treatments with initial concentrations ranging from 0.095 to 12.16 µg/L. Fish were observed for 48 h, and changes in concentrations of 6PPD‐quinone were monitored throughout the experiment. No mortalities or substantial changes in behavior were recorded in either Atlantic salmon or brown trout. This provides an important first step in assessing effects of 6PPD‐quinone on these economically and culturally highly important species. *Environ Toxicol Chem* 2022;41:3041–3045. © 2022 The Authors. *Environmental Toxicology and Chemistry* published by Wiley Periodicals LLC on behalf of SETAC.

## INTRODUCTION

The compound 6PPD‐quinone is an oxidation product of *N*‐(1,3‐dimethylbutyl)‐*N*′‐phenyl‐*p*‐phenylenediamine (6PPD; Tian et al., 2021), which is an additive in tire rubber and constitutes a substantial proportion of vehicle tires (0.4%–2%; Babbit, 2010). Wear and tear from tires contributes substantially to environmental pollution, with per capita estimates of 0.23–4.7 kg/year and a global average of 0.81 kg/year (Kole et al., 2017). McIntyre et al. (2021) showed that tire wear particles induced acute mortality in adult coho salmon (*Oncorhynchus kisutch*), whereas the closely related chum salmon (*Oncorhynchus keta*) were asymptomatic. McIntyre's research was followed by that of Tian et al. (2021), who identified 6PPD‐quinone as the primary factor inducing the mortality in coho salmon, with a median lethal concentration (LC50) of 0.79 µg/L. This LC50 was slightly later found to be substantially lower (0.095 µg/L) as a result of improved analytical methods and the availability of commercial reference standards of 6PPD‐quinone (Tian et al., 2022).

Following these findings, toxicity testing has been conducted with untreated urban roadway runoff, previously documented to contain 6PPD‐quinone, showing lethal effects on juvenile rainbow trout (*Oncorhynchus mykiss*), coho salmon, chinook salmon (*Oncorhynchus tshawytscha*), but not sockeye salmon (*Oncorhynchus nerka*; French et al., 2022). Furthermore, using 6PPD‐quinone solutions, Brinkmann et al. (2022) showed 6PPD‐quinone toxicity in brook trout (*Salvelinus fontinalis*) and rainbow trout. The 6PPD‐quinone toxicity in rainbow trout has also been confirmed by Di et al. (2022). Lack of response to 6PPD‐quinone at environmentally relevant concentrations has also been documented in Artic char (*Salvelinus alpinus*) and white sturgeon (*Acipenser transmontanus*; Brinkmann et al., 2022), zebrafish (*Danio rerio*) larvae (Hiki et al., 2021; Varshney et al., 2022), Japanese medaka (*Oryzias latipes*; Hiki et al., 2021) and a cyprinid fish endemic to China (*Gobiocypris rarus*; Di et al., 2022).

It should also be noted that 6PPD (the parent compound of 6PPD‐quinone) has been shown to induce acute lethality at high concentrations (more than 100 µg/L) in zebrafish, Japanese medaka, and *Gobiocypris rarus*; Di et al., 2022; Hiki et al., 2021; Varshney et al., 2022).

The apparent species‐specific toxicity of 6PPD‐quinone complicates risk assessment of this chemical for species other than the ones that have been tested. In vitro studies of rainbow trout gill and liver cells indicate that 6PPD‐quinone toxicity might be driven by a tissue‐specific disruption of mitochondrial respiration (Mahoney et al., 2022), whereas exposure of juvenile coho salmon to roadway runoff suggests blood–brain barrier disruption as a potential mechanism (Blair et al., 2021). Until the precise mechanism(s) that cause the toxic effect have been established, and the physiological differences that prevent the toxic effect in some species have been identified, a species‐by‐species approach will be necessary to assess toxicity. We present the first results from acute toxicity testing of 6PPD‐quinone in Atlantic salmon (*Salmo salar*) and brown trout (*Salmo trutta*).

## MATERIALS AND METHODS

### Exposure experiment

Toxicity testing was conducted in the period March 26–28, 2022 at the Norwegian Institute for Nature Research (NINA) Aquatic Research Station located in Rogaland, western Norway (58°50′N, 6°E). The experiment was carried out over 48 h in static water. The fish used in the experiment were alevins of Atlantic salmon (*S. salar*) and brown trout (*S. trutta*) from the research station stock. Hatching date, defined as the date when 50% of the eggs in a batch had hatched, was the March 15 for the Atlantic salmon and February 22 for the brown trout.

For both Atlantic salmon and brown trout, a total of eight 6PPD‐quinone treatments were conducted, with two controls. Solutions of 6PPD‐quinone (0.095, 0.19, 0.38, 0.76, 1.52, 3.04, 6.08, and 12.16 µg/L) were prepared at the Norwegian Veterinary Institute using fish medium matching the water chemical properties (see just below) of the water at the research station where the fish had been kept prior to the experiment. Water from the research station originated from a nearby lake, with a deep intake (13 m) and was ozone treated, filtered (mesh 0.1 mm; Bernoulli), UV treated, and oxygenated to 100% saturation. Water quality was monitored monthly, and important water quality parameters from March 2022 were as follows: temperature 4.9 °C, pH 6.9, turbidity 0.36 FNU, conductivity 7.3 mS/m, Ca^2+^ 4.4 mg/L, Mg^2+^ 1.3 mg/L, total N 0.79 mg/L, and total organic carbon 3.0 mg/L. The two controls consisted of fish medium and water from the research station. All data including full water chemistry are freely available (see *Data Availability Statement*).

Prior to exposure, all 6PPD‐quinone solutions, fish medium, and control water were aerated using an aquarium air pump for 3 h. The mean O_2_ concentration was 12.8 mg/L (range 12.1–13.5 mg/L). The control water from the research station was oxygenated prior to entering the hatchery and had a higher O_2_ concentration (14.2 mg/L). The experiment was conducted in the same room, and treatments were placed in the same type of hatchery chambers as the fish were hatched in, minimizing changes in environment. Fish were gently transferred from hatchery tanks to the treatments using a plastic cup. To reduce the stress involved in catching exactly 10 fish, some treatments included more than 10 fish (range 10–13). The wet weights of 10 Atlantic salmon alevins and 10 brown trout alevins were 1.1067 and 0.7634 g, respectively. Water was quickly drained from the cup using a fine mesh, before releasing the fish into treatments of 1 L. Previously unused and thoroughly cleaned glass containers (approximately 1.5 L) were used for the treatments. The temperature during the treatments was kept as close as possible to the temperature experienced by the fish prior to the experiment (4.9 °C) by placing bags of ice in the center of each chamber containing treatments. Throughout the experiment, temperature and oxygen content in treatments were monitored using a Handy Polaris 2 (Oxyguard), and pH measurements were done on 30‐ml aliquots using a PHM92 device (Radiometer). Fish were observed every 3–7 h throughout the experiment, and fish that were not actively moving were gently touched on the caudal peduncle to produce a reaction. Water samples were collected in preprepared probes (see *Probe design and sample preparation* section) after 3, 10, 24, 36, and 48 h for analysis of 6PPD‐quinone concentrations.

### Chemicals

Acetonitrile and water for instrumental analyses and methanol were Optima liquid chromatography–mass spectrometry (LC–MS) grade; calcium dichloride dihydrate (99%) and magnesium sulfate heptahydrate (99%) were acquired from Fisher Scientific. Formic acid (for LC‐MS, 97.5%–98.5%; LiChropur™) and sodium bicarbonate (99.5% or more; ReagentPlus) were from Sigma‐Aldrich (Merck). The 6PPD‐quinone and ^13^C_6_‐6PPD‐quinone were acquired from Cambridge Isotope Laboratories. Dust‐free paper wipes were from Kimtech (Kimberly‐Clark).

### Preparation of fish media and test solutions

Fish water media was prepared to match the chemical properties of the water at the NINA Aquatic Research Station. Calcium dichloride, magnesium sulfate, and sodium bicarbonate were dissolved in Milli‐Q water with final concentrations of 0.31, 0.028, and 0.30 mM, respectively. The 6PPD‐quinone was dissolved in acetone (100 mg/L), and 1 ml of this solution was poured into a 2‐L glass bottle. The solvent was evaporated using a flow of nitrogen, and the residue was dissolved in fish medium aided by stirring for 60 min and sonication for 10 min. The accurate concentration of this stock solution was determined by LC–MS/MS as outlined in the *LC–MS/MS instrument conditions* section. The 6PPD‐quinone stock solution was used for preparation of test solutions with target concentration of 12.2, 6.08, 3.04, 1.52, 0.760, 0.380, 0.190, and 0.095 µg/L by serial dilutions. All solutions were kept at 5 °C prior to the experiment.

### Probe design and sample preparation

Quantification of 6PPD‐quinone in the fish medium during the experiment was achieved by sampling of medium aliquots into prepared probes. Probes were designed to compensate uncertainty caused by decomposition of 6PPD‐quinone in water solution. Each probe consisted of a 1.5‐ml polypropylene tube to which a 2 × 2‐cm piece of dust‐free paper wipe had been added, which was the carrier substrate for the internal standard. The internal standard (1 ng of ^13^C_6_‐6PPD‐quinone) had previously been deposited on the paper carrier by adding 20 µl of a 50‐µg/L working solution in acetone. After evaporation of acetone (~5 min), the probes were closed and stored at 5 °C until use. During the experiment, 1‐ml aliquots of fish media were collected and transferred to individual probes. Probes were kept at 5 °C, and were analyzed 5 days after the exposure trial.

### LC–MS/MS instrument conditions

Samples were analyzed using an Agilent 6470A triple‐stage quadrupole mass spectrometer, equipped with a Jet Stream electrospray interface operated in positive ion mode, and coupled to an Agilent 1290 Infinity II LC System (Agilent Technologies). Probes were centrifuged at 22 °C and 15 000 *g* for 10 min, and 500‐µl aliquots were transferred into high‐performance liquid chromatography (HPLC) vials. One microliter of each sample was injected onto an Agilent Eclipse Plus C18 RRHD ultra(U)HPLC column (3 × 50‐mm, 1.8‐µm particle size; Agilent Technologies). Mobile phase A was 0.1% formic acid in water, and mobile phase B was 0.1% formic acid in acetonitrile. The column was kept at 30 °C and eluted using a linear gradient as follows: start with 20% B and rising to 90% over 4 min, followed by isocratic elution (90% B) for 1 min and equilibration with 20% B for another 1 min. The flow rate was 0.6 ml/min across the entire LC run. Two transitions were monitored for both 6PPD‐quinone and its isotopically labeled internal standard: 6PPD‐quinone, *m/z* 299.1 → 187.3 (collision energy [CE] 31 eV) and *m/z* 299.1 → 77.3 (CE 77 eV); ^13^C_6_‐6PPD‐quinone, *m/z* 305.1 → 193.3 (CE 31 eV) and *m/z* 305.1 → 83.3 (CE 77 eV) for quantification and confirmation, respectively. Other instrument parameters were gas temperature: 250 °C; gas flow: 5 L/min; nebulizer: 45 psi; sheath gas temperature: 350 °C; sheath gas flow: 10 L/min; capillary voltage: 3500 kV; and nozzle voltage 500 V. Quantification of 6PPD‐quinone was achieved by linear 10‐point calibration using a set of standards dissolved in methanol. The concentration of ^13^C_6_‐6PPD‐quinone in the calibrants was 1 µg/L, and concentrations of 6PPD‐quinone were chosen as 0.02, 0.05, 0.1, 0.2, 0.5, 1, 2, 5, 10, and 20 µg/L. Calibrant solutions were kept at −20 °C between analyses. The instrumental data were processed using the Agilent MassHunter Quantitative Analysis software Ver B.08.00.

### Method evaluation

The retention time for 6PPD‐quinone was 3.6 min, which allowed separation of the target compound from other matrix constituents. The run time for one LC‐MS/MS analysis was 7 min. No interfering peaks were observed when we analyzed negative or blank samples, confirming the method's selectivity. The trueness of the method was evaluated as apparent recovery of 6PPD‐quinone by comparing the peak‐area ratios between 6PPD‐quinone and the internal standard (^13^C_6_‐6PPD‐quinone) for two quality controls (QCs) with those analyzed without the carrier. Quality controls were prepared by addition of 6PPD‐quinone water solution to fish media, with final concentrations of 0.4 and 2.5 µg/L for QC1 and QC2, respectively. The apparent recovery of 6PPD‐quinone was found to be 96% for QC1 and 97% for QC2 (*n* = 3, relative standard deviation more than 10%). The QCs were also used for determination of the limits of detection (LOD) and quantification (LOQ). The LOD was calculated at a signal/noise ratio of 3:1, and found to be 0.006 µg/L, whereas the LOQ was 0.020 µg/L based on a signal/noise of 10:1 (Kryuchkov et al., in prep.). Ten‐point calibration curves (0.020–20.0 µg/L) showed excellent linearity (*r* ^2^ = 0.9999).

## RESULTS AND DISCUSSION

**Figure 1:**
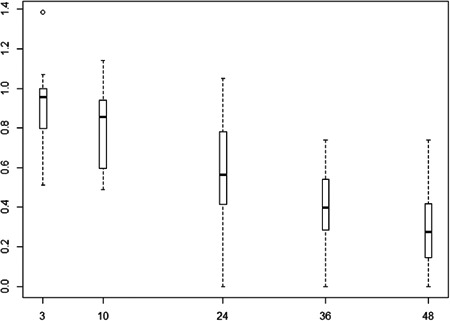
Boxplot of relative concentration of 6PPD‐quinone in relation to initial concentration (*y*‐axis) sampled during the experiment (hours after start of experiment, *x*‐axis). The bottom and top of the box indicate the 25th and 75th percentiles (i.e., the boxes include the middle 50% of observations), the whiskers span to the most extreme data point if more than 1.5 times the interquartile range, and the bold horizontal line represents the median value.

No mortalities or abnormal behavior were recorded for either Atlantic salmon or brown trout during the experiment. Average (±SD) water temperature during the experiment was 4.7 (±0.51) °C and never diverged more than 1.7 °C from the temperature at which fish were kept prior to the experiment. Oxygen levels remained high during the experiment; the average O_2_ concentration after 24 h was 10.6 mg/L (range 10.0–11.1), and after 48 h it was 10.3 mg/L (range 9.77–10.7). The pH was stable throughout the experiment, with an average pH of 6.4 (range 6.0–6.7). Concentrations of 6PPD‐quinone decreased substantially during the 48 h (Figure 1), and were on average 28% of the initial concentrations after 48 h. The 6PPD‐quinone concentration in two treatments with initial concentrations of 12.16 µg/L decreased to 5.75 and 8.98 µg/L after 48 h. This finding shows that the experienced concentrations were high enough to conclude that these species do not exhibit mortality well above environmentally relevant concentrations of 6PPD‐quinone (Cao et al., 2022; Kryuchkov et al., in prep.; Tian et al., 2022). The reduction of 6PPD‐quinone concentrations can likely be ascribed to both water‐chemical and biological factors. The reported half‐life in dechlorinated tap water (33 h at 23 °C; Hiki et al., 2021) differs substantially from the half‐life reported in ultrapure water and untreated river water (12.8–16.3 days at 25 °C; Di et al., 2022), indicating a strong effect of the water chemistry and its constituents. Furthermore, uptake of 6PPD‐quinone by the fish will reduce concentrations, and adsorption to laboratory equipment is also expected for the relatively lipophilic compound. The previous studies showing toxicity of 6PPD‐quinone were conducted using older juvenile stages or adults, and at higher temperatures (~10 °C). Because the mechanism that causes 6PPD‐quinone toxicity has not yet been determined, we would advise caution in generalizing our findings to other conditions (temperatures and water chemistry) and other life stages of these species until further studies have been conducted. Synergistic negative effects of 6PPD (the parent compound of 6PPD‐quinone) with other contaminants have been demonstrated in rotifers (Klauschies & Isanta‐Navarro, 2022). Such potential for synergistic effects with 6PPD‐quinone might complicate ecological risk assessments substantially.

**Figure 2:**
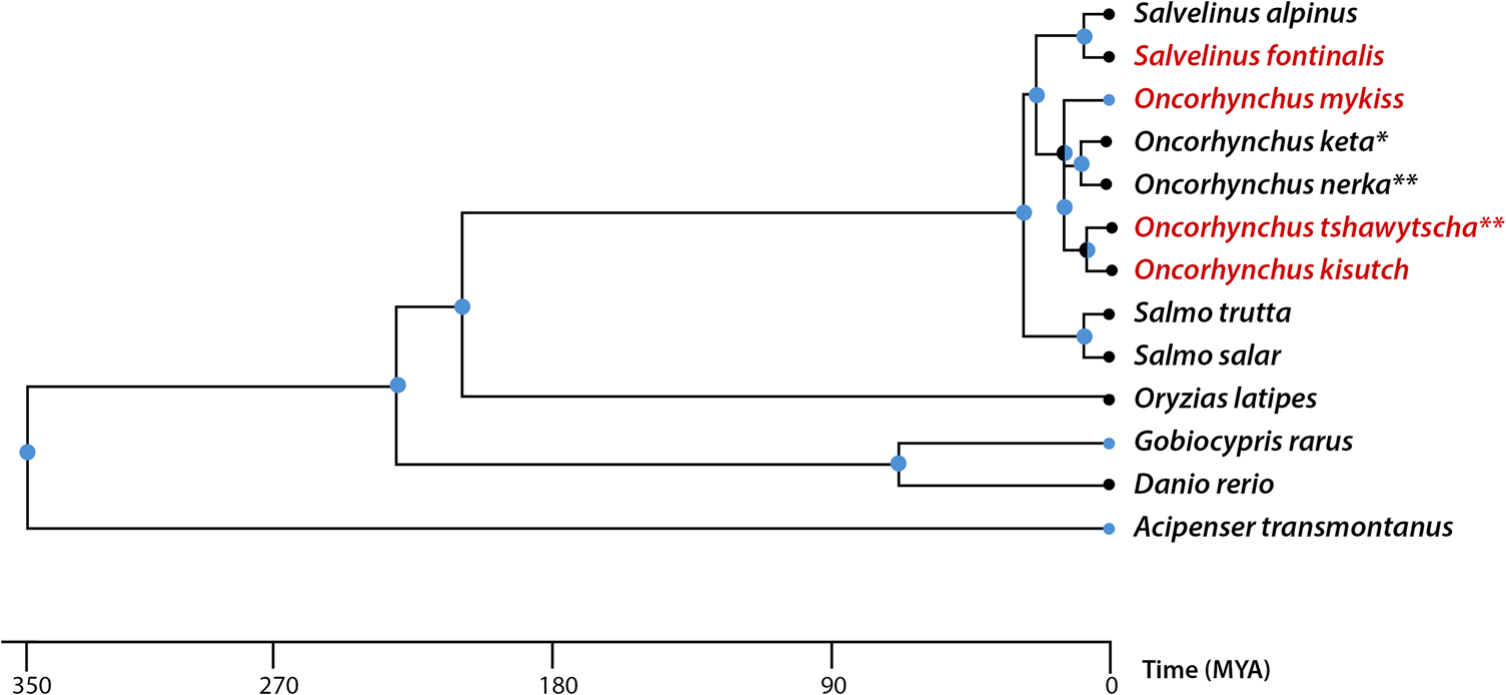
Phylogenetic tree of the fish species that have been tested for 6PPD‐quinone toxicity. Presumed toxicity is indicated for three species; *tested with tire wear particle leachate, **tested with urban roadway runoff. Species names of tolerant species are in black and sensitive species in red (phylogeny from timetree.org).

To our knowledge, this is the first toxicity test conducted on species of the *Salmo* genus. Both Atlantic salmon and brown trout are economically and culturally important species, and thus any negative effects from 6PPD‐quinone on these species would be especially relevant for conservation and management. Of the currently tested fish species, toxicity of 6PPD‐quinone has not been shown outside the *Oncorhynchus* and *Salvelinus* genera (Figure 2). Also, 6PPD‐quinone was not toxic to the crustaceans *Daphnia magna*, and *Hyalella azteca*, even at the maximum water solubility of the chemical (Hiki et al., 2021).

The widespread occurrence of 6PPD‐quinone and its high toxicity to some species constitutes a serious challenge for management and conservation of natural populations of the sensitive species. However, the apparent variation in susceptibility also emphasizes that 6PPD‐quinone may have potential as a species‐selective biocide. All the species shown to be sensitive to 6PPD‐quinone occur as invasive species outside their natural range (Crawford & Muir, 2008). Given that toxicity is restricted to these species, and found not to negatively impact other aquatic organisms, 6PPD‐quinone could potentially be used to selectively remove these species without endangering endemic species.

Our study demonstrates that alevins of Atlantic salmon and brown trout showed no toxicity to 6PPD‐quinone under the tested conditions. We strongly recommend that further studies be conducted using older life stages of these species and that they also include different water chemistries before making general conclusions regarding the toxicity of 6PPD‐quinone. A precise understanding of the chemical and physiological mechanisms causing toxicity coupled with knowledge of environmental occurrence and fate of 6PPD‐quinone will be necessary for assessing environmental risk of the chemical.

## Author Contributions Statement

**Anders Foldvid**: Conceptualization; Funding acquisition; Investigation; Methodology; Project administration; Resources; Supervision; Writing—original draft; Writing—review & editing. **Fedor Kryuchkov**: Conceptualization; Formal analysis; Investigation; Methodology; Resources; Validation; Writing—original draft; Writing—review & editing. **Roar Sandodden**: Conceptualization; Funding acquisition; Methodology. **Silvio Uhlig**: Conceptualization; Formal analysis; Investigation; Methodology; Resources; Validation; Writing—original draft; Writing—review & editing.

## Ethical Statement

The experiment used alevins of Atlantic salmon and brown trout, this is a life history stage that does not require approval for experiment from the Norwegian Food Safety Authority.

###  

This article has earned both an Open Data and an Open Materials badge for making publicly available the digitally shareable data necessary to reproduce the reported results. The data are available at https://osf.io/9gpxc/?view_only=20f883462d214413b91f9d232dc54fa4. Learn more about the Open Practices badges from the Center for Open Science: https://osf.io/tvyxz/wiki.

## Data Availability

All data and supporting information is openly available at https://osf.io/9gpxc/?view_only=20f883462d214413b91f9d232dc54fa4.

## References

[etc5487-bib-0001] Babbit, R. O. (2010). The Vanderbilt rubber handbook (14th ed.). R. T. Vanderbilt.

[etc5487-bib-0002] Blair, S. I. , Barlow, C. H. , & McIntyre, J. K. (2021). Acute cerebrovascular effects in juvenile coho salmon exposed to roadway runoff. Canadian Journal of Fisheries and Aquatic Science, 78, 103–109. 10.1139/cjfas-2020-0240

[etc5487-bib-0003] Brinkmann, M. , Montgomery, D. , Selinger, S. , Miller, J. G. P. , Stock, E. , Alcaraz, A. J. , Challis, J. K. , Weber, L. , Janz, D. , Hecker, M. , & Wiseman, S. (2022). Acute toxicity of the tire rubber‐derived chemical 6PPD‐quinone to four fishes of commercial, cultural, and ecological importance. Environmental Science & Technology Letters, 9, 333–338. 10.1021/acs.estlett.2c00050

[etc5487-bib-0004] Cao, G. , Wang, W. , Zhang, J. , Wu, P. , Zhao, X. , Yang, Z. , Hu, D. , & Cai, Z. (2022). New evidence of rubber‐derived quinones in water, air, and soil. Environmental Science & Technology, 56, 4142–4150. 10.1021/acs.est.1c07376 35316033 PMC8988306

[etc5487-bib-0005] Crawford, S. S. , & Muir, A. M. (2008). Global introductions of salmon and trout in the genus *Oncorhynchus*: 1870–2007. Reviews in Fish Biology and Fisheries, 18, 313–344.

[etc5487-bib-0006] Di, S. , Liu, Z. , Zhao, H. , Li, Y. , Qi, P. , Wang, Z. , Xu, H. , Jin, Y. , & Wang, X. (2022). Chiral perspective evaluations: Enantioselective hydrolysis of 6PPD and 6PPD‐quinone in water and enantioselective toxicity to *Gobiocypris rarus* and *Oncorhynchus mykiss* . Environment International, 166, 107374. 10.1016/j.envint.2022.107374 35780684

[etc5487-bib-0007] French, B. F. , Baldwin, D. H. , Cameron, J. , Prat, J. , King, K. , Davis, J. W. , McIntyre, J. K. , & Scholz, N. L. (2022). Urban roadway runoff is lethal to juvenile coho, steelhead, and Chinook salmonids, but not congeneric sockeye. Environmental Science & Technology Letters, 9, 733–738. 10.1021/acs.estlett.2c00467 36118959 PMC9476652

[etc5487-bib-0008] Hiki, K. , Asahina, K. , Kato, K. , Yamagishi, T. , Omagari, R. , Iwasaki, Y. , Watanabe, H. , & Yamamoto, H. (2021). Acute toxicity of a tire rubber‐derived chemical, 6PPD quinone, to freshwater fish and crustacean species. Environmental Science & Technology Letters, 8, 779–784. 10.1021/acs.estlett.1c00453

[etc5487-bib-0009] Klauschies, T. , & Isanta‐Navarro, J. (2022). The joint effects of salt and 6PPD contamination on a freshwater herbivore. Science of the Total Environment, 829, 154675. 10.1016/j.scitotenv.2022.154675 35314241

[etc5487-bib-0010] Kole, P. J. , Löhr, A. J. , Van Belleghem, F. G. A. J. , & Ragas, A. M. J. (2017). Wear and tear of tyres: A stealthy source of microplastics in the environment. International Journal of Environmental Research and Public. Health, 14, 1265. 10.3390/ijerph14101265 29053641 PMC5664766

[etc5487-bib-0011] Kryuchkov, F. , Foldvik, A. , Sandodden, R. , & Uhlig, S. (In Prep.). Quantification of 6PPD‐quinone in tunnel wash waters and several relevant environmental samples.

[etc5487-bib-0012] Mahoney, H. , da Silva Junior, F. C. , Roberts, C. , Schultz, M. , Ji, X. , Alcaraz, A. J. , Montgomery, D. , Selinger, S. , Challis, J. K. , Giesy, J. P. , Weber, L. , Janz, D. , Wiseman, S. , Hecker, M. , & Brinkmann, M. (2022). Exposure to the tire rubber‐derived contaminant 6PPD‐quinone causes mitochondrial dysfunction in vitro. Environmental Science & Technology Letters, 9, 765–771. 10.1021/acs.estlett.2c00431

[etc5487-bib-0013] McIntyre, J. K. , Prat, J. , Cameron, J. , Wetzel, J. , Mudrock, E. , Peter, K. T. , Tian, Z. , Mackenzie, C. , Lundin, J. , Stark, J. D. , King, K. , Davis, J. W. , Kolodziej, E. P. , & Scholz, N. L. (2021). Treading water: Tire wear particle leachate recreates an urban runoff mortality syndrome in coho but not chum salmon. Environmental Science & Technology, 55, 11767–11774. 10.1021/acs.est.1c03569 34410108

[etc5487-bib-0014] Tian, Z. , Gonzalez, M. , Rideout, C. A. , Zhao, H. N. , Hu, X. , Wetzel, J. , Mudrock, E. , James, C. A. , McIntyre, J. K. , & Kolodziej, E. P. (2022). 6PPD‐quinone: Revised toxicity assessment and quantification with a commercial standard. Environmental Science & Technology Letters, 9, 140–146. 10.1021/acs.estlett.1c00910

[etc5487-bib-0015] Tian, Z. , Zhao, H. , Peter, K. T. , Gonzalez, M. , Wetzel, J. , Wu, C. , Hu, X. , Prat, J. , Mudrock, E. , & Hettinger, R. (2021). A ubiquitous tire rubber–derived chemical induces acute mortality in coho salmon. Science, 371, 185–189. 33273063 10.1126/science.abd6951

[etc5487-bib-0016] Varshney, S. , Gora, A. H. , Siriyappagouder, P. , Kiron, V. , & Olsvik, P. A. (2022). Toxicological effects of 6PPD and 6PPD quinone in zebrafish larvae. Journal of Hazardous Materials, 424, 127623. 10.1016/j.jhazmat.2021.127623 34742612

